# Editorial: Protein-RNA interplay-regulated signaling in stem cells and cancer

**DOI:** 10.3389/fcell.2023.1304817

**Published:** 2023-10-16

**Authors:** Xichen Bao, Xiaoxing Li, William K. K. Wu, Yanquan Zhang, Liang Zhou

**Affiliations:** ^1^ Laboratory of RNA Molecular Biology, Guangdong Provincial Key Laboratory of Stem Cell and Regenerative Medicine, CAS Key Laboratory of Regenerative Biology, GIBH-CUHK Joint Research Laboratory on Stem Cell and Regenerative Medicine, Guangzhou Institutes of Biomedicine and Health, Chinese Academy of Sciences, Guangzhou, China; ^2^ Institute of Precision Medicine, The First Affiliated Hospital of Sun Yat-sen University, Guangzhou, China; ^3^ Department of Anaesthesia and Intensive Care, State Key Laboratory of Digestive Diseases, Li Ka Shing Institute of Health Sciences, The Chinese University of Hong Kong, Hong Kong, China; ^4^ Department of Toxicology and Cancer Biology, College of Medicine, University of Kentucky, Lexington, KY, United States; ^5^ Department of Toxicology, Guangdong Provincial Key Laboratory of Tropical Disease Research, School of Public Health, Southern Medical University, Guangzhou, China

**Keywords:** epitranscriptome, RNA modifications, stem cells, cancer, transcriptional regulatinon

The RNA-protein interplay has been proven to be essential for precise regulation of both RNA and protein, which has many implications in various biological processes including stem cell maintenance, differentiation, carcinogenesis and so on (Ye and Blelloch, 2014; Pereira et al., 2017). More specifically, RNA binding proteins (RBPs) have been shown to regulate RNA metabolism ranging from transcription, modification, processing, nuclear export, translation to RNA decay (Hentze et al., 2018; He et al., 2023). On the other hand, RNA molecules have also been shown to regulate RBP functions such as protein stability, enzymatic activity, translocations (Ni et al., 2019; Deng et al., 2020; Huppertz et al., 2022). In this Research Topic, we focused mainly on the RNA-protein interplay in specific physiological (e.g., stem cell and neurogenesis) and pathological (e.g., cancer or heart diseases) contexts, with emphasis on the RNA modifications in cancer.

Mammalian development begins with a fertilized egg. This process is associated with proper organ or tissue formation with precise cell fate determination. Deng et al. reviewed how RNA degradation machinery selectively clears specific transcripts during early cell fate determinations including maternal-to-zygotic transition, pluripotency maintenance, as well as somatic cell reprogramming Deng et al. Moreover, Chan et al. reviewed the function of many RBPs (e.g., CPEB3, FXR2) in later-stage adult neurogenesis. The authors also discussed that RBPs are involved in many aspects of neurogenesis including cell proliferation, migration, and differentiation (Chan et al., 2022).

The dysregulation of RNA-protein interplay leads to diseases including neuronal diseases, learning defects as discussed by Chan and colleagues (Chan et al., 2022). Moreover, its dysfunction can also cause many other diseases including various cancers and heart diseases. In this Research Topic, Xu et al. demonstrated that 13 out of 14 5-methylcytosine (m^5^C)-associated RBPs are generally amplified in ovarian cancer, suggesting a direct role of m^5^C and its associated RBPs in cancer development or therapy. Moreover, the authors established a prognostic prediction model based on several of the m^5^C regulators including ALYRER, NOP2, and TET2 for overall survival prediction Xu et al.. Besides m^5^C modification and its associated RBPs, other types of RNA modifications are also involved in cancer development or cancer therapy through various mechanisms (Deng X. et al., 2023). Liu W. et al. conducted a comprehensive analysis on one of the N6-methyladenosine (m^6^A) readers, YTHDF2, across various cancers. They showed that YTHDF2 might be a biomarker for tumor detection or prognostic analysis Liu W. et al. Chen et al. summarized m^6^A and its associated protein partners in regulating cancer stemness properties Chen et al. Cancer stem cells are a small subpopulation of cancer cells with the capacity of self-renewal or contributing to the spread of cancer cells, the understanding of m^6^A and its associated RBPs in cancer stemness provides potential therapeutic strategies for future cancer treatment.

ADAR-mediated A-to-I editing is a more traditional RNA modification to modulate RNA structure, coding sequences on RNAs, which plays critical roles in regulating tumorigenesis and has many implications in therapeutics or prognosis (Jiang et al., 2017; Liu J. et al.) discussed both the editing-dependent and editing-independent roles of ADAR1 in mature mRNA and non-coding RNA (e.g., microRNA, long non-coding RNA and circular RNA) during cancer development. They also discussed the A-to-I editing events in intron or untranslated region, and their effect on translation or mRNA stability in cancer Liu J. et al.

Besides the roles of RNA-protein interplay in cancer, Liu et al. explored the m^6^A pattern in heart failure with preserved ejection fraction (HFpEF), compared with the HFpEF plus exercise mouse model. They showed that HFpEF plus exercise mouse model displays higher total m^6^A level and reduced FTO level. Further investigation demonstrated that FTO can promote myocyte apoptosis, myocardial fibrosis, and hypertrophy, which could be a therapeutic target for HFpEF Liu K. et al.

In this Research Topic, we also included two papers focusing on the specific cellular processes regulated by RNA-protein interplay. Chen et al. summarized the enhancer RNA and its partners in regulating gene transcription. The authors also discussed the involvement of RNA modifications and liquid phase condensates in gene transcription Chen et al. Cheung et al. reviewed the roles of RNA modifications and their associated proteins in the regulation of ferroptosis, a new type of programmed cell death. Moreover, they also discussed their potential applications for therapeutic manipulation in cancer Cheung et al.

Altogether, our Research Topic included relevant work or reviews on RNA-protein interplay in both development and diseases. We hope that our topic will be helpful for improving the understanding of RNA-protein interplay at both cellular and molecular level.
